# Insights into the Use of C-Reactive Protein as a Diagnostic Index of Disease Severity in COVID-19 Infections

**DOI:** 10.4269/ajtmh.20-0473

**Published:** 2020-06-25

**Authors:** Lawrence A. Potempa, Ibraheem M. Rajab, Peter C. Hart, Jose Bordon, Rafael Fernandez-Botran

**Affiliations:** 1 College of Pharmacy, Roosevelt University, Schaumburg, Illinois;; 2 Washington Health Institute, Washington, District of Columbia;; 3 Department of Pathology and Laboratory Medicine, University of Louisville, Louisville, Kentucky

## Abstract

Approximately 20% of patients infected with SARS-CoV-2 (COVID-19) develop potentially life-threatening pathologies involving hyperinflammation, cytokine storm, septic shock complications, coagulation dysfunction, and multiple organ failure. Blood levels of the prototypic acute phase reactant, C-reactive protein (CRP), which is hepatically synthesized and released in response to interleukin-6 stimulation, is markedly elevated in patients with COVID-19. Markedly high CRP levels correlate with poor prognosis for survival. Insights into CRP structure–function relationships have uncovered both pro- and anti-inflammatory isoforms that may be used to monitor the extent of tissue damage associated with COVID-19 pathologies and prognoses. Herein, rationale is given for interpretation of CRP blood levels as a simple, rapid, and cost-effective way to assess disease severity and help guide therapeutic options in COVID-19 patients.

Since December 2019, the SARS-CoV-2 (COVID-19) pandemic has continued to extend over most of the world, infecting several millions of people and causing more than 250,000 deaths to date (https://coronavirus.jhu.edu/map.html, accessed on May 6, 2020). There has been an intense focus on technologies to diagnose infections by the virus and to assess the prevalence of the infection. Equally important, there has been an unprecedented effort to identify and develop drugs, vaccines, and technologies to treat and prevent further infections as well as to assess disease severity and reduce mortality in affected patients. The purpose of this article was to offer an insight into the potential use of C-reactive protein (CRP) levels as a diagnostic index of disease severity and a guide for therapeutic options in COVID-19–infected patients.

Approximately 20% of patients infected with SARS-CoV-2 (COVID-19) develop potentially life-threatening pathologies involving acute inflammation, cytokine storm, septic shock complications, coagulation dysfunction, metabolic acidosis, hypoxia, and multiple organ failure.^1^ Consistent blood markers in afflicted patients are normal to low white cell counts and elevated interleukin-6 (IL-6) levels which, among its many activities, signal the liver to increase synthesis and secretion of CRP.^2–5^

C-reactive protein is a widely used diagnostic marker primarily used to assess ongoing inflammation. It is a key protein of the acute phase response, appears in blood within 6–10 hours of any tissue damaging event, has a plasma half-life of 19 hours, and is produced without a memory response. Although its appearance in blood is associated with ongoing inflammatory responses, it does not selectively accumulate into any tissue or organ even in patients known to have ongoing, prominent inflammation. Furthermore, its fractional catabolic rate is independent of its plasma concentration, indicating its rapidly changing blood concentration more nearly reflects on its synthesis rate and not its consumption rate.^6^

In normal healthy individuals, baseline CRP levels in blood are reported to be less than 10 μg/mL,^7,8^ with measured CRP below this threshold described as “high sensitivity CRP” values. In situations of tissue trauma or disease, CRP blood levels can increase 10- to 100-fold within 10–72 hours of the inciting cause (i.e., described as “conventional CRP” values), with higher levels correlating with disease severity. Although levels between 10 and 50 μg/mL are diagnostic of an ongoing inflammatory response that can be either acute or chronic, CRP levels above 100 μg/mL in any disease are diagnostic of fulminant inflammation and are associated with poor disease prognosis. The U.S. Department of Health and Human Services guidelines for interpreting the diagnostic significance of CRP describe the difference between conventional and high sensitivity values and provides guidance only for conventional CRP levels.^9^

C-reactive protein levels have been used to differentiate viral from bacterial infections.^10^ In uncomplicated viral infections, CRP levels increase minimally to ∼ 20 μg/mL, whereas in bacterial infections, levels increase to > 40 μg/mL. These levels reflect on the degree of tissue involvement and can help diagnose confounding complications. Zhang et al.^11^ reported in 140 hospitalized COVID-19 patients with confirmed SARS-CoV-2 infection, CRP levels varied from 28.7 μg/mL in non-severe disease (*n* = 82; range, 9.5–52.1 μg/mL) to 47.6 μg/mL in severe disease (*n* = 56; range, 20.6–87.1 μg/mL) (see Table 1). In previously studied respiratory viral infections, patients infected with the avian flu strain H7N9 (that emerged in 2013) had mean CRP levels of 84.0 μg/mL. Of note, mean CRP levels in patients who died were 129.6 μg/mL compared with 63.0 μg/mL in those who survived.^12^ Higher CRP levels in viral infections may be diagnostic of a concurrent bacterial infection and have been used to help decide whether infected patients should be placed on antibiotic therapy.^13–16^

**Table 1:** Serum CRP levels correlate with the severity of COVID-19 and mortality

Reference	Normal values (μg/mL)	All COVID-19 cases	Non-severe COVID-19	Severe COVID-19	Mortality
Zhang et al.^11^	0–3	34.7	28.7	47.6	n/a
Guan et al.^19^	< 10	60.7	56.4	91.1	n/a
Richardson et al.^20^	0–4	130 (64–269)	n/a	n/a	24.5%
Liu et al.^21^	CRP > 41.8 had greater risk of mortality				

n/a = not available.

Severe cases of COVID-19 develop a hyperinflammatory response, leading to a pathological dysfunction of innate host defense systems. Complications include cytokine release syndrome (i.e., cytokine storm) and multiple organ failure. Fundamental to all pathologies is tissue damage, which, when severe enough, leads to organ dysfunction and death. As CRP blood levels correlate with activation of the acute phase response, they represent a simple, rapid, and cost-effective way to assess the degree of tissue damage ongoing in that patient at the time of measurement. Tan et al.^17^ reported significantly increased CRP levels in initial stages of COVID-19 disease, with levels correlated with the severity of disease and the degree of internal tissue pathologies as measured using CT technology. C-reactive protein levels that rapidly return to baseline are an index of resolving tissue damage as may occur with favorable patient responses to treatments. At the early stage of COVID-19, CRP levels were positively correlated with lung lesions. C-reactive protein levels could reflect disease severity and should be used as a key indicator for disease monitoring.^18^ Table 1 summarizes recent data highlighting the correlation between CRP levels and severity of COVID-19 infection.

Insight into a biological role for CRP as part the inflammatory response has evolved with the discovery and understanding that CRP can exist in at least two distinct conformational isoforms with opposing effects on inflammation. C-reactive protein that is measured in plasma (including that measured in COVID-19 patients) is a very soluble, non-covalently associated cyclic pentameric protein that has weak anti-inflammatory bioactivities. This pentameric protein, however, can be induced to non-proteolytically dissociate from individual subunits which, when separated, undergoes a pronounced rearrangement into an isoform with distinctive solubility, antigenicity, and bioactivity. The dissociation of the pentamer occurs on activated cell membranes (e.g., on endothelial cells and platelets) so that as the CRP subunit undergoes conformational change, it enters the bilayer lipid rafts. The membrane-associated CRP monomer stimulates strong pro-inflammatory intracellular signaling pathways that amplify the acute phase inflammatory response and include activation of pathways to recruit and activate leukocyte responses^22,23^ and induce platelet aggregation.^24^ In early phases of acute inflammation, the conversion of the soluble pentamer into the membrane-bound monomer is efficient, and acute inflammation is amplified. As the expression of the membrane-bound isoform slows down, the levels of pentameric CRP measured in blood increase, acting as a marker for an unamplified acute phase response and dysregulated inflammation. Hence, higher CRP levels in blood are diagnostic of extensive tissue damage and a pathological inflammatory response. From the point of view of the pathophysiology of the COVID-19 infection and its complications, it is tempting to speculate that the membrane-associated monomeric CRP isoform may play a role, particularly promoting pro-inflammatory and procoagulant manifestations.

In COVID-19 infections, CRP levels that are minimally elevated (e.g., between 10 and 20 μg/mL) can be diagnostic of mild viral disease. Therapeutic considerations might focus on ways to control viral infectivity and replication using viral neutralization concepts (antibodies/vaccines and receptor blockers), or inhibiting viral-specific enzymes (e.g., remdesivir). COVID-19 patients with moderate elevations of CRP levels (e.g., > 20–40 μg/mL) may harbor some level of (reversible) tissue damage associated with the natural response to combating the viral disease. If these levels are measured relatively early in disease progression, before a cytokine storm response, these levels may suggest a confounding bacterial infection or more significant tissue involvement in disease. Therapeutic considerations may include, in addition to viral neutralization, the use of antimicrobials and immunomodulation using interferon therapies or biologic agents that can neutralize pro-inflammatory factors (e.g., tumor necrosis factor-alpha (TNFα, interleukin-1 (IL-1, and IL-6 inhibitors). COVID-19 patients with significantly elevated CRP levels (e.g., > 100 μg/mL) more readily reflect advanced tissue damage and pathologies associated with cytokine storm, coagulation abnormalities, and multiple organ failure. Such high CRP levels would correlate with a life-threatening prognosis.

Of note, some success in treating patients in severe cytokine storm has been measured using tocilizumab, an anti-IL-6 receptor antagonist biotherapeutic. As IL-6 is the key cytokine signaling the synthesis and release of CRP and because IL-6 (and CRP) levels do correlate with the severity of COVID-19 disease, the relationship among these factors and the relevance of each in the establishment and progression of COVID-19 infection needs evaluation. Xu et al.^25^ reported CRP levels decreased markedly from 75 ± 67 to 38.13 + 54.21 μg/mL 1 day after tocilizumab therapy. Levels continued to fall to 10.61 ± 13.79 μg/mL 3 days after therapy, and to 2.72 ± 3.6 μg/mL 5 days after therapy. In another clinical trial not causally related to COVID-19 infection, a patient given chimeric antigen receptor T cell (CAR-T) therapy developed a severe cytokine storm response like that seen in severe COVID-19 infection. Whereas use of steroid and vasopressor was ineffective, treatment with tocilizumab to block the IL-6 response was found to rapidly resolve the life-threatening complications occurring in that patient.^4^

The role of CRP and its distinct isoforms in the pathogenesis of COVID-19 infection remains to be studied. However, the accumulating evidence of their correlation supports the notion of the use of CRP levels as a diagnostic index of the severity of COVID-19 infection. Moreover, it may be possible to develop a point-of-care CRP test for patients with fever or other basic clinical symptoms that is noninvasive, highly predictive of health outcomes, and which may inform clinical decision-making.
